# The Adoption of Smoking and Its Effect on the Mortality Gender Gap in Netherlands: A Historical Perspective

**DOI:** 10.1155/2015/370274

**Published:** 2015-07-26

**Authors:** Fanny Janssen, Frans van Poppel

**Affiliations:** ^1^Population Research Centre, University of Groningen, Groningen, Netherlands; ^2^Netherlands Interdisciplinary Demographic Institute (NIDI/KNAW), The Hague, Netherlands

## Abstract

We examine in depth the effect of differences in the smoking adoption patterns of men and women on the mortality gender gap in Netherlands, employing a historical perspective. Using an indirect estimation technique based on observed lung cancer mortality from 1931 to 2012, we estimated lifetime smoking prevalence and smoking-attributable mortality. We decomposed the sex difference in life expectancy at birth into smoking-related and nonsmoking-related overall and cause-specific mortality. The smoking epidemic in Netherlands, which started among men born around 1850 and among women from birth cohort 1900 onwards, contributed substantially to the increasing sex difference in life expectancy at birth from 1931 (1.3 years) to 1982 (6.7 years), the subsequent decline to 3.7 years in 2012, and the high excess mortality among Dutch men born between 1895 and 1910. Smoking-related cancer mortality contributed most to the increase in the sex difference, whereas smoking-related cardiovascular disease mortality was mainly responsible for the decline from 1983 onwards. Examining nonsmoking-related (cause-specific) mortality shed new light on the mortality gender gap and revealed the important role of smoking-related cancers, the continuation of excess mortality among women aged 40–50, and a smaller role of biological factors in the sex difference than was previously estimated.

## 1. Introduction

It is well known that there are clear sex differences in mortality, with women generally having lower mortality and thus higher life expectancy than men [1]. In many western countries this female advantage in mortality started to increase in the early decades of the 20th century [2, 3] and rose rapidly during the 1950s and 1960s. In the final decades of the 20th century, however, the female advantage started to decline (e.g., [4]). In northwestern Europe, the female advantage in life expectancy at birth is currently about four to five years. In eastern Europe, however, the female advantage continues to be large, at around 10 years [5, 6].

Differences in health-related behaviour/lifestyle have been shown to play an important role in explaining sex differences in mortality. Although biological differences account for about 25% of the sex difference in life expectancy in western European countries [7], the remainder of the gap can in large part be explained by social and behavioural factors (e.g., [3]). Because the role of socioeconomic factors seems to have been limited in western Europe in the late 20th century [8], the majority of the gap appears to be attributable to the earlier adoption among men than women of risky health behaviour [9, 10], including smoking [11, 12], motor vehicle driving [13, 14], substance use, alcohol consumption, and extreme sports [15].

The fact that men are generally more prone to risk-taking than women [16] can be related to their sensation-seeking personality, to the “risk as value” hypothesis, and to the restrictions placed on risk-taking by the cultural context. The size of the gender gap in the adoption of risky behaviour varies as a function of a culture's restrictiveness, the norms for appropriate gender role behaviour [16], and time. Women, in general, however, tend to follow men some decades later in the adoption of risky behaviour. Waldron has offered several hypotheses regarding how the changed position of women in society led to the adoption of risky health behaviour among women [17]. With the rise in women's labour force participation, women increasingly became exposed to occupational hazards and job stresses, and their levels of independence and personal income grew, which made women more prone and more able to adopt risky health behaviour. But also, the increase in women's labour force participation may have indirectly changed females roles and led to a general liberalisation of norms concerning women's behaviour. Moreover, the interaction of socioeconomic, cultural, and material conditions with fundamental aspects of traditional gender roles may have contributed to the delayed adoption of risky behaviour among women.

An important health-related behaviour with a clear impact on sex differences in mortality is smoking (e.g., [3, 5, 7, 18–21]). Although estimates of the contribution of smoking vary according to the time period and the country studied, McCartney et al. recently estimated that smoking-related causes of death explained 40%–60% of the gender gap in all-cause mortality in Europe in 2003–2005 [5]. Similarly, based on an analysis of 44 European countries over the period 1950/55 to 2005/2009, Luy and Wegner-Siegmundt showed that smoking explained on average more than 40% of the sex difference in life expectancy in 21 of these countries, most of them in western Europe [7].

However, previous studies on the contribution of smoking to the sex gap in mortality were not able to depict the full smoking epidemic because of their limited time range. In addition, most of these studies examined the contribution of smoking to sex differences in all-cause mortality, without exploring the underlying causes of death. Given that smoking is an important, but not the only contributing, factor in the gender gap, it would be helpful to look beyond the role of smoking, as Luy and Wegner-Siegmundt have also recently recommended [7]. Examining the remaining sex difference when the role of smoking is eliminated is one of the most obvious ways of doing this.

In this paper we will analyse in depth the effect of sex differences in the adoption of smoking on the gender gap in mortality in Netherlands. In conducting our analysis, we will (i) adopt a historical perspective, (ii) identify by which causes of death smoking mainly contributed to both the increase and the decrease in the sex difference, and (iii) evaluate the sex difference in nonsmoking-related mortality. Netherlands is particularly interesting given the enormous high smoking prevalence among Dutch men in the past and the relatively late onset of the smoking epidemic among Dutch women.

## 2. Materials and Methods

To assess the contribution of smoking- and nonsmoking-related mortality to the sex difference in life expectancy at birth for Netherlands, we first obtained life table data, all-cause mortality, and exposure data from the Human Mortality Database for 1900–2009 by year, sex, and single year of age and complemented these data based on death and population numbers from Statistics Netherlands for 2010–2012 [25].

We estimated, for each year, lifetime smoking prevalence and the share of all deaths that can be attributed to smoking (= smoking-attributable mortality fractions) by five-year age groups and sex using the indirect Peto et al., 1992, method [26]. This methodology uses observed lung cancer mortality rates as a proxy for lifetime smoking prevalence, using the fact that almost all lung cancer mortality is due to smoking and combines this prevalence with relative risks of dying from smoking to assess smoking-attributable mortality, thereby taking into account the fact that smoking affects not only lung cancer mortality but also other causes of death.

The necessary lung cancer mortality deaths (ICD3: 47ab; ICD4: 47abc; ICD5: 47abc; ICD6-7: 162-163; ICD8: 162; ICD-9: 162; ICD-10: C33-C34) by age (40–44,45–49,…, 80+) and sex were available from 1931 onwards and were obtained directly from publications by Statistics Netherlands for 1931–1949 [27], through WHOSIS (http://www.who.int/healthinfo/statistics/mortality_rawdata/en/) (update July 2012) for 1950–2009 and from Statistics Netherlands for 2010–2012 [25].

As a first step in the indirect estimation of smoking-attributable mortality, we obtained, for each year and sex, estimates of the proportion of the population exposed to smoking during their lifetime, which we label here as lifetime smoking prevalence by five-year age groups (*p*_*i*_).

We used the lung cancer mortality data for this purpose but controlled for lung cancer mortality that is not due to smoking, by comparing, for each sex, the obtained age-specific lung cancer mortality rates (*r*_*i*_^*T*^) with the smoothed age-specific lung cancer rates of the smokers (*r*_*i*_^SM^) and the never-smokers (*r*_*i*_^NS^) in the American Cancer Study (ACS) CPS-II [26]. More specifically, lifetime smoking prevalence by age group (*p*_*i*_) is calculated for each year and sex by

(1) $$\begin{array}{c} p_{i}=\frac{r_{i}^{T}-r_{i}^{\text{NS}}}{r_{i}^{\text{SM}}-r_{i}^{\text{NS}}}. \end{array}$$

Negative results were converted to zeros, while results larger than one were converted to one [28]. We graphed the lifetime smoking prevalence by birth cohort, age, and sex.

As a second step, we estimated for each sex and year the age-specific proportions of deaths attributable to smoking (SAF_*i*_) using the formula of the population attributable fraction: SAF_*i*_ = *p*_*i*_(RR_*i*_ − 1)/(*p*_*i*_(RR_*i*_ − 1) + 1), where *p*_*i*_ reflects the obtained estimates of the lifetime smoking prevalence by age group and RR_*i*_, the relative risks of dying from smoking by age group. The RR_*i*_ were calculated directly from the all-cause mortality rates for smokers and never-smokers in the ACS CPS-II study [26] and were subsequently smoothed by applying a second-degree polynomial. We reduced the excess risk by 30% to control for the exposure of smokers to other risk factors [28]; that is, we applied 1 + (RR_*i*_ − 1)∗0.7.

The Peto et al. methodology we used to indirectly estimate smoking-related mortality [26] assumes that the relative risk of dying from smoking—and the difference in the risk faced by males and females—stays the same over time. This assumption can certainly be debated. The methodology is, however, frequently used, and the estimates have been shown to be largely similar to recent regression-based methods [29, 30]. As these regression-based techniques can only be applied to all-cause mortality from 1950 onwards, they were not useful for our more historical perspective, in which we also examine cause-specific mortality, and, indirectly, estimated lifetime smoking exposure.

To assess the role of smoking in more detail, we compared, for each single age, the relative sex differences in mortality for all-cause mortality with the sex differences in nonsmoking-related mortality using the so-called two shaded contour maps [31]. To make the contour map of the ratio of male to female nonsmoking-related mortality, we obtained nonsmoking-related mortality rates for each sex and single year of age (*x*) by multiplying the all-cause mortality rates (*M*_*x*_^*T*^) by one minus the smoking-attributable mortality fractions by single year of age; that is, *M*_*x*_^NS^ = *M*_*x*_^*T*^∗(1 − SAF_*x*_). For this purpose, we turned the earlier obtained smoking-attributable mortality fractions by five-year age groups into single-year values using least squares linear regression, with the value for ages 80+ applied to all single ages 83 to 110+.

To examine by which causes of death smoking contributed the most to both the increase and the decrease in the sex difference, we obtained cause-of-death data and divided the cause-specific mortality into smoking-related and nonsmoking-related mortality.

The cause-of-death data for 65 cause-of-death groups were obtained from Wolleswinkel-van den Bosch for 1901–1992 [22] and from Statistics Netherlands for 1993–2012. Based on these 65 causes and their classifications [23], six main cause-of-death groups were constructed: infectious diseases, cancers, cardiovascular diseases, chronic respiratory diseases, external causes, and others. See (1) of the Appendix for the ICD-9 codes used. The cause-of-death data were available by age (age groups 0, 1–4, 5–14, 15–19, 20–49, 50–64, 65–79, and 80+) and sex.

To divide the cause-specific deaths into smoking-related and nonsmoking-related mortality, we used RRs of dying from smoking for the selected causes of death which were based on the unsmoothed cause-specific mortality rates for smokers and nonsmokers from the ACS-CPS II study [26] by sex and by ages 35–39,…, 75–79, 80+. We smoothed these values by age by means of a second polynomial. Note, however, that we had to use different starting ages for the regressions, and in some cases we had to set a RR smaller than one to one. See (2) of the Appendix. Again, we reduced the excess risk of the different causes of death by 30% to control for confounding, as suggested by Ezzati and Lopez [28].

To obtain the smoking-attributable mortality fractions (originally 35–39,…, 75–79, 80+) for the right age groups (20–49, 50–64, 65–79, 80+), they were regrouped using weights based on mortality for the different age groups for the specific cause of death in 2012 [25].

For infectious disease we did not distinguish between smoking- and nonsmoking-related mortality, because of a lack of information on the RR of dying from smoking for infectious disease. We calculated “other smoking-related mortalities” by subtracting cause-specific smoking-related mortality from total smoking-related mortality.

Using the Arriaga decomposition technique, we decomposed the sex difference in life expectancy at birth into the main contributing causes of death and age groups [32], thereby distinguishing for each cause of death the smoking-related mortality and the nonsmoking-related mortality.

Our approach heavily relies on the quality of the cause-of-death information. For lung cancer mortality, which is very important for the estimation of smoking-attributable mortality, the data quality is generally high because the disease has a straightforward diagnosis. Despite possible changes in diagnosing lung cancer over time, it should be noted that in Netherlands in the late 1920s the cancer statistics obtained already a lot of attention by many specialists [33]. To overcome quality issues for the remaining causes of death, we used large cause-of-death groups that were proven to be consistent over time, according to the meticulous reclassification approach by Wolleswinkel-van den Bosch [23].

## 3. Results and Discussion

### 3.1. The Smoking Epidemic in Netherlands

In Netherlands, in the absence of national smoking prevalence data before the 1950s, the start of the smoking epidemic can be estimated using information from the cigar and cigarette industry. The industrial production of cigars and the automation of the production process of cigarettes started around 1880–1885. Between 1914 and 1920 the cigarette industry expanded and cigarettes started to become consumer goods [34].

Data for 1907 in Amsterdam indicate that, among 25,000 schoolboys aged 6–12, more than half smoked, and 74% of the boys aged 11-12 smoked. In other large cities, but also in the countryside, comparable figures were observed [35]. Around this point in time, especially in countries not involved in WWI, the only concerns expressed about smokers in health textbooks were about young male smokers [36]. Also, cigarette marketing campaigns focused only on males [37]. Before WWII, smoking among ordinary women in Netherlands was stigmatised [38].

**Figure 1:**
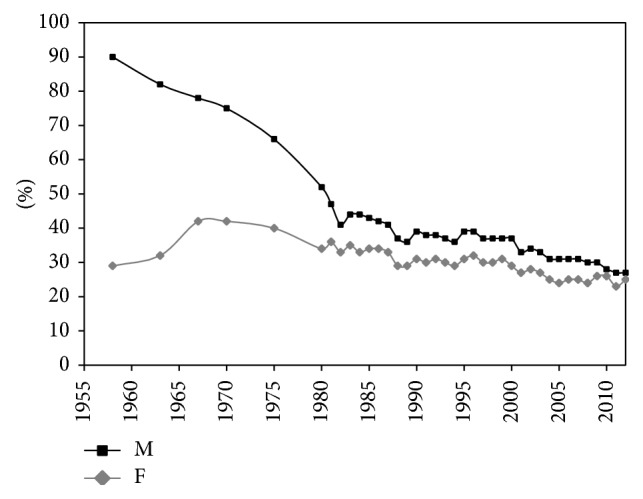
Smoking prevalence (15+) by sex, Netherlands, 1958–2012. Source data: Stivoro (2013) [24]; M = males; F = females.

After 1950, more information on the sex differences in smoking became available. A study among schoolchildren in Amsterdam in 1957 showed that 47% of the boys and 11% of the girls had smoked more than once [35]. The first survey on smoking in 1958 indicated that smoking prevalence was 90% among adult men and 29% among adult women [24] (Figure 1). Whereas among adult men the smoking prevalence was around 90% in all age groups, among women smoking prevalence was highest in the age group 20–34, at 46% [24]. Gadourek observed that it were especially the better educated women who smoked and who consumed more cigarettes [39]. It was a combination of changes in the role and status of women and the promotion by the tobacco industry of smoking as a symbol of emancipation that made smoking by women socially acceptable [40].

Whereas the percentage of men who smoked dropped to 27% in 2012, the percentage among women increased rapidly, rising to 42% in 1967. After 1975 (still 42%), the share declined slowly, falling to around 25% in 2012 (Figure 1). Whereas almost all men started smoking in a period in which the health risks of smoking were not yet known, smoking did not start to become popular among Dutch women until the 1950s–1960s, when the dangers of smoking were already known [35]. As a result the smoking prevalence among women peaked at much lower levels (42%) as compared to men (90%).

The peak in smoking prevalence among women around 1965–1970 is reflected in a peak in estimated past smoking intensity about 35 years later ((3) of the Appendix), which seems to indicate that the peak in smoking prevalence among men occurred a few years before 1958.

**Figure 2:**
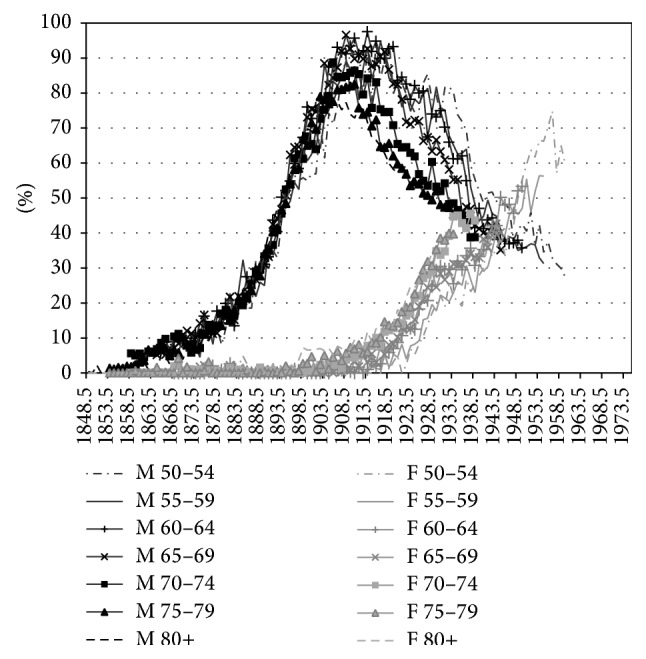
Estimated lifetime smoking exposure by age and sex, by birth cohort, Netherlands, 1931–2012. M = males; F = females.

Figure 2 shows the differences between men and women in terms of their estimated lifetime smoking exposure by birth cohort. Also here it can clearly be observed that the smoking epidemic started earlier among Dutch men than among Dutch women. A sharp rise in lifetime smoking exposure can be seen among men born as early as 1850. But among women, lifetime smoking exposure started to increase only from the birth cohort 1900 onwards. Men born between 1895 and 1910 clearly had the highest lifetime smoking exposure, which was demonstrated earlier as well [41]. Also, the increasing tobacco consumption among women born after 1930, who reached adulthood after 1950, was observed before [42].

Comparing the estimated lifetime smoking intensities by age and cohort with the observed smoking prevalence data by five-year age groups and birth cohort (based on data from calendar year 1988 onwards) in (4) of the Appendix, it can be observed that (i) a clear decline in smoking prevalence for men born between 1905 and 1935 occurred, although the observed smoking prevalence levels are much lower than those estimated by means of the past smoking intensities, which is likely because the smoking prevalence data only include current smokers and not previous smokers, (ii) the smoking prevalence for adult men born from 1935 onwards is quite stable at levels around 40%, and (iii) for women the increase for birth cohorts from 1905 up to 1955 and the decline thereafter are clearly in line with the estimated past smoking intensities, although the observed smoking prevalence levels are slightly lower than the estimated smoking intensities. Note, however, that the original sources behind the observed prevalence data differ for calendar years 1958, 1963–1975, and 1979–2012. Only from calendar year 1980 onwards the data are based on a sample size of 10,000 to 20,000 [24]. Also, the smoking prevalence data do not reflect the dosage of smoking, an important factor when estimating the effects of smoking on mortality and health, whereas our indirect estimation of lifetime smoking exposure does.

### 3.2. Effect of Smoking on the Mortality Gender Gap

**Figure 3:**
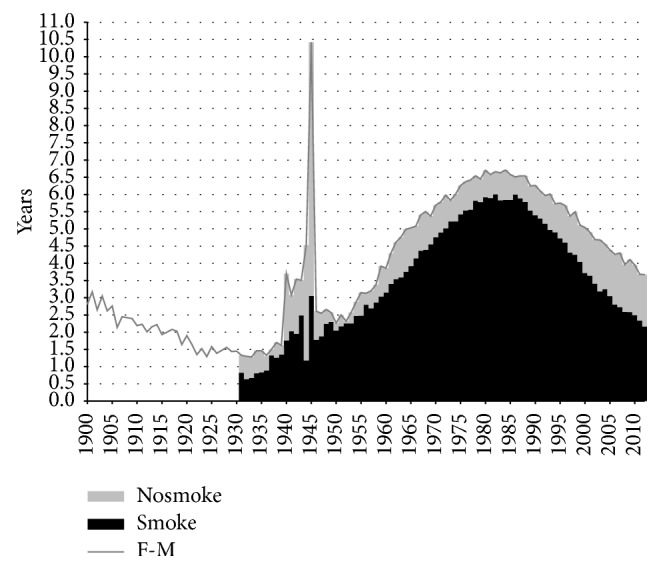
Contribution of smoking- and nonsmoking-related mortality to the difference in life expectancy at birth between men and women, in years, Netherlands, 1900–2012. Nosmoke = nonsmoking-related mortality; smoke = smoking-related mortality. M = males; F = females.

Smoking contributed substantially to the sex difference in life expectancy (Figure 3). The female advantage in life expectancy at birth in Netherlands declined from around three years in 1900 to around 1.5 years in the 1920s. From 1931 onwards, when the difference in life expectancy was 1.3 years, the female advantage began to increase substantially. This trend, which lasted until around 1982, resulted in a maximum difference in life expectancy of 6.7 years. From 1982 onwards, the female advantage underwent a strong decline. By 2012, life expectancy was 3.7 years higher for Dutch women than for Dutch men. Whereas smoking contributed just 0.8 years in 1931, this number went as high as 6.0 years in 1982 and 1986, though it subsequently declined to 2.2 years in 2012. The relative contribution of smoking to the sex difference in life expectancy was the highest in 1952, when it reached 98%. The share declined thereafter, falling to 59% by 2012.

**Figure 4:**
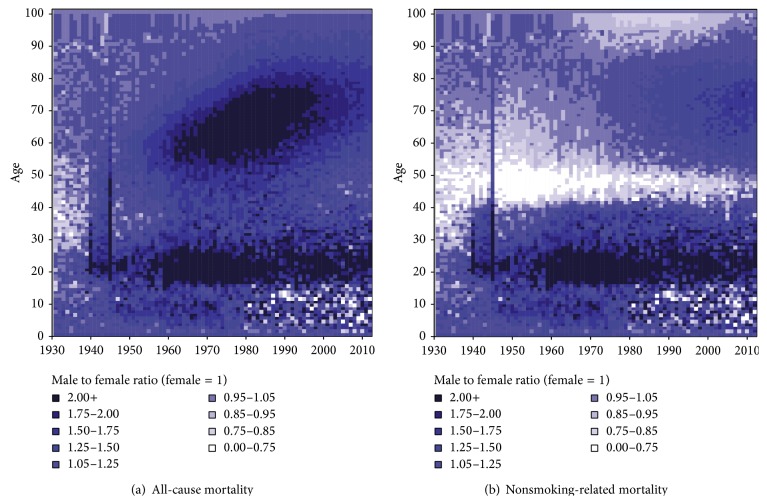
Comparison of the ratios of male-to-female mortality rates for all-cause mortality versus nonsmoking-related mortality, 1931–2012.

When we examined the underlying ratio of male-to-female all-cause mortality rates using a contour map, we found two distinct patterns of male excess mortality (Figure 4(a)). First, a strong increase in excess mortality among men aged 16–26 years occurred since the 1940s. Second, an increase in excess mortality among men occurred at ages above 55 after 1950, reflecting high mortality rates among male cohorts born between 1892 and 1905. In addition, we can see that women actually had slightly higher mortality than men, particularly in the age group 30–40, up to the 1930s. From 1980 onwards, girls had even higher death rates than boys for certain ages up to age of 17.

Whereas accidents and suicide are frequently mentioned as being the main source of excess mortality among men around age 20 [3] and maternal mortality is cited as being the primary cause of excess mortality among women in the age group 30–40 [43], smoking is clearly behind the excess mortality among older men after 1950 as it reflects the high lifetime smoking exposure among Dutch men born between 1895 and 1910. And indeed, when we examine the contour map for nonsmoking-related mortality, the cohort pattern is no longer visible (Figure 4(b)).

**Figure 5:**
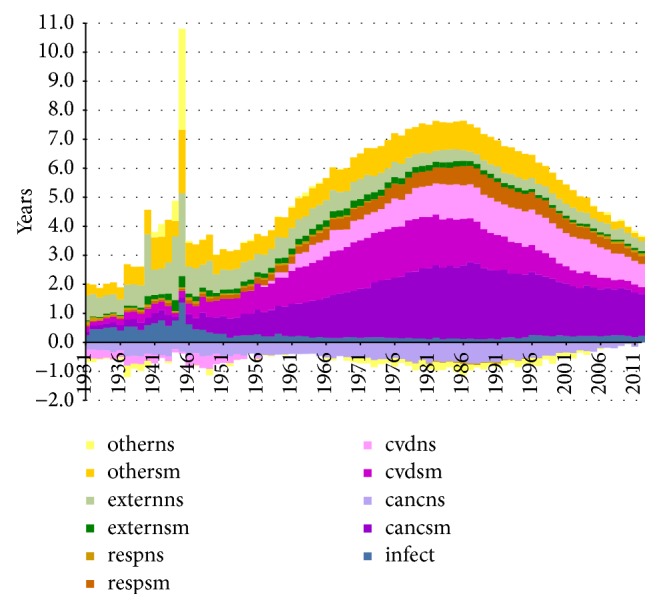
Contribution of smoking- and nonsmoking-related cause-specific mortality to the differences in life expectancy at birth between men and women, in years, Netherlands, 1931–2012. infect = infectious disease mortality; cancsm = smoking-related cancer mortality; cancns = nonsmoking-related cancer mortality; cvdsm = smoking-related cardiovascular disease mortality; cvdns = nonsmoking-related cardiovascular disease mortality; respsm = smoking-related respiratory disease mortality; respns = nonsmoking-related respiratory disease mortality; externsm = smoking-related external disease mortality; externns = nonsmoking-related external disease mortality; othersm = other smoking-related mortalities; otherns = other nonsmoking-related mortalities.

When we examine the causes of death and the age groups that are behind the trends in the sex difference in life expectancy, we find that the increase in life expectancy between 1931 and 1950 can already be attributed to smoking-related cancer and cardiovascular disease mortality, even though external mortality and infectious diseases are the most important causes of death in that period (Figure 5, Table 1). Note as well that, in this period, there was actually excess mortality among women for nonsmoking-related cancers—probably due to breast cancer and gynaecological cancers [44]—and nonsmoking-related cardiovascular disease, the latter being in line with observed slightly higher rates of overall cardiovascular disease mortality for Dutch women as compared to Dutch men in this period.

The rapid increase in the sex difference after 1950 is largely attributable to cardiovascular disease and cancer in the age group 65–79. Smoking-related cancer mortality was the main contributor, and the total contribution of cancer mortality was made up of a very strong effect of smoking-related cancer mortality offset by a negative contribution of higher mortality from nonsmoking-related cancers among women. Smoking-related cardiovascular disease mortality made the largest contribution to cardiovascular disease mortality, although the contribution of nonsmoking-related cardiovascular disease mortality also increased from 1955 onwards.

The decline in the sex difference in life expectancy from 1983 onwards seems to mainly be due to smoking-related cardiovascular disease mortality in the age group 50–64. The sex difference in smoking-related cardiovascular disease mortality greatly diminished from 1983 to 2012 and is currently only marginal. The contribution of nonsmoking-related cardiovascular disease mortality stayed around one year.

Additional analysis of the effect of smoking on the sex difference in remaining life expectancy at age 50 (see (5) of the Appendix) revealed that the trend in the sex difference in remaining life expectancy at age 50 is similar to the one observed for life expectancy at birth, although the sex difference is slightly smaller at age 50 than at birth. For remaining life expectancy at age 50, the increase in the sex difference can almost completely be attributed to the increase in the sex difference in smoking-related mortality. After 1983, the decline in the sex difference in remaining life expectancy at age 50 is driven by the decline in the sex difference in smoking-related mortality but is slightly counterbalanced by the increase in sex difference for nonsmoking-related mortality, similar to what we observed for life expectancy at birth. This seems to imply that it is mainly smoking-related mortality from age of 50 onwards that is behind the sex difference in life expectancy and that—when controlled for age-gender specific survival factors at younger ages—smoking plays an even larger role in the increase in the gender gap up to 1983.

The comparison of the observed role of smoking in the gender gap in mortality with other studies is not straightforward, as it very much depends on the period examined, the characteristics of the country examined—like the time of the onset of the smoking epidemic and the popularity of smoking relative to, for example, alcohol [3]—and the overall extent of the mortality difference between the sexes. Similarly, we should be careful when we try to generalise a certain estimate of the role of smoking in the sex difference in mortality. We should also note that estimates for a single country can differ due to the methodology used. For example, Luy and Wegner-Siegmundt observed for Netherlands over the period 1955/1959–2005/2009 an average gender gap of 5.5 years, of which 62.5% (3.44 years) is due to smoking [7]. We find, however, an average contribution of smoking of 4.4 years out of 5.4 years (80%) over the same period. Additional analysis showed that this substantial difference can be mainly explained by the 50% reduction in excess risk to account for the confounding used in the original Peto-Lopez method [26], as applied by Luy and Wegner-Siegmundt and by our 30% reduction in excess risk using the more recent insights by Ezatti and Lopez [28]. The difference in results with Valkonen and van Poppel can as well be linked to the same issue, but also to the methodology to assess the role of smoking in the sex difference. Valkonen and van Poppel estimated that smoking contributed 3.8 years (72%) to the sex differences in life expectancy at age 35 in Netherlands in 1970–1974. In the period 1985–89, the contribution was 3.2 years (53%) [20]. However, our results indicate that smoking contributed 84% to the sex difference in life expectancy in 1972 and 90% in 1987. A comparison of the sex difference in life expectancy for nonsmoking-related mortality with the sex difference in life expectancy for all-cause mortality (their approach) is, however, bound to result in different outcomes than a decomposition (our methodology), because life expectancy for nonsmoking-related mortality is calculated based on the assumption that all smoking-related mortality would be eliminated.

Our results for smoking-attributable all-cause mortality proved to be similar to the recent results from the Global Burden of Disease (GBD) study in 2010, which applied the indirect Peto-Lopez method combined with epidemiological data to estimate lung cancer mortality in nonsmokers. This seems to validate both our approach and theirs.

A comparison of the trend over time in the sex difference in life expectancy proved to be more straightforward. The trend we observed for Netherlands since 1950 was similar to the trend observed in countries such as United Kingdom, Denmark, Norway, and Sweden [45]. For these countries as well smoking has been found to play an important role, albeit a smaller one than for Netherlands [7, 20]. In the middle of the 1960s, the prevalence of male smokers in Netherlands was much higher than that observed in the other European countries and at that time even one of the highest in the world. In the same period, the smoking prevalence among women was rather small in Netherlands, and lagged far behind the percentages found in the United Kingdom, where women's roles were affected by experiencing WWI, and Denmark, which was also one of the forerunner countries in terms of female smoking [46, 47].

### 3.3. Remaining Sex Differences in Life Expectancy at Birth

When examining the trend in the gender gap in nonsmoking-related mortality (Figure 3), we can see that from 1983 onwards the advantage of women in terms of nonsmoking-related mortality increased from 0.8 to 1.5 years. This slight increase is in line with the overall divergence between the sexes in nonsmoking-related mortality that Pampel observed for 21 high-income nations combined over the period from 1975 to 2000 [48]. Examining Table 1 shows the importance of an increasing sex difference in nonsmoking-related respiratory disease mortality and the disappearance of the male advantage for nonsmoking-related cancer mortality. Additional analysis revealed that the increases in the contribution of nonsmoking-related mortality over this period mainly took place at ages 50 and over. Behavioural factors, such as the larger uptake of preventive health behaviour among women than men and the more frequent uptake of new risky behaviour among men than among women, are postulated to be behind these trends [9, 10, 48, 49]. The recent stabilisation in the sex difference in nonsmoking-related mortality from approximately 2006 onwards could point to a new phase in which gender differences in preventive health behaviour are disappearing, although it first should be established whether this is a long-term and international phenomenon.

When we examine the contour map for nonsmoking-related mortality, next to the disappearance of the excess mortality among older men after 1950, some additional interesting patterns are brought forward which were previously offset by the effect of smoking, that is, (i) a very large amount of excess mortality among women aged 40–50, (ii) excess mortality among women aged 90+ between 1970 and 2010, and (iii) a small amount of excess mortality among men around ages 65–85, which emerged in 1970 and increased and expanded to the age group 50–90 over time. The large amount of excess nonsmoking-related mortality among women aged 40–50, particularly before 1980, seems to be largely a continuation of excess all-cause mortality among women aged 30–40 from 1850 to 1910 and among women aged 30–50 from 1910 to 1940, which can in large part be explained by maternal mortality [43]. For the higher ages, it is very likely that higher mortality among women from breast cancer and gynaecological cancers, such as cancer of the uterus and cancer of the ovaries, also plays a role [44]. This indeed seems in line with the, at that period, observed amount of excess mortality among women in nonsmoking-related cancer mortality.

Part of the remaining difference in nonsmoking-related mortality is due to biological factors. Previous estimates of the sex difference in life expectancy caused by biological factors amount to around two years at birth [50, 51], to maximum two years at age 25 [52], or, more generally, to approximately 25% when the sex difference in life expectancy is between 1.5 and 6 years [7]. This latter would indicate that for Netherlands the biological effect on the sex difference in life expectancy would be 1.66 years in 1983 and 0.92 years in 2012.

However, the remaining difference we found was less than the suggested 25% from 1948 to 1999 (e.g., for 1983, 0.82 years = 12%) and was larger than the suggested 25% from 2000 onwards (e.g., for 2012, 1.51 years = 41%). Because the overall sex difference largely depends on the scale of the smoking epidemic, which varies considerably across countries, it would seem that assessing the effect of biological factors based on nonsmoking-related mortality would give us a better estimate than based on all-cause mortality, at least for western European countries until the end of the 20th century.

Overall, however, the remaining sex difference amounts to between 0.1 years (1952) and 1.6 years (2007), which seems to indicate that biological factors play a smaller role than was previously estimated, given that other lifestyle factors also still have an effect.

## 4. Conclusion

The smoking epidemic in Netherlands, which started among men born around 1850 and among women from birth cohort 1900 onwards, contributed substantially to the increasing sex difference in life expectancy at birth from 1931 (1.3 years) to 1982 (6.7 years), the subsequent decline to 3.7 years in 2012, and the high excess mortality among Dutch men born between 1895 and 1910. Smoking-related cancer mortality was the main contributor to the increase in the sex difference, whereas smoking-related cardiovascular disease mortality was mainly responsible for the decline from 1983 onwards. Examining nonsmoking-related (cause-specific) mortality shed new light on the mortality gender gap. It revealed the continuation of excess mortality among women aged 40–50. But it also suggested that biological factors may play a smaller role in the sex difference than was previously estimated.

Assessing the effect of biological factors for nonsmoking-related cause-specific mortality would be an important step forward. To do so, it is important to control for the role of smoking in the gender gap in the general population when examining the biological effect and to estimate the biological effect for the different causes of death.

## Figures and Tables

**Figure 6:**
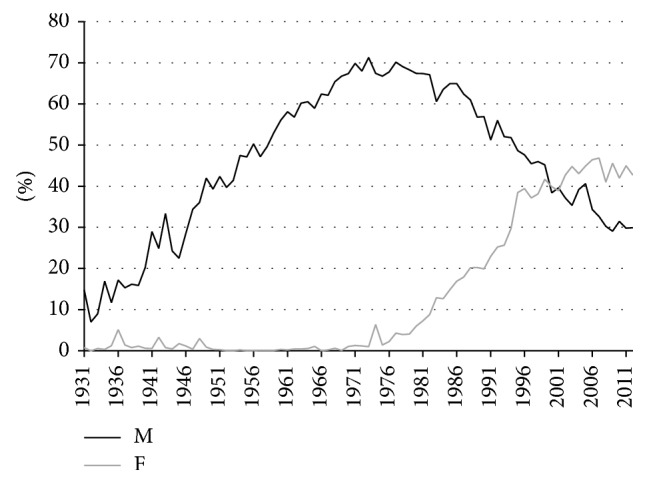
Estimated past smoking exposure aged 35 and over, Netherlands, 1931–2012, by sex. M = males; F = females.

**Figure 7:**
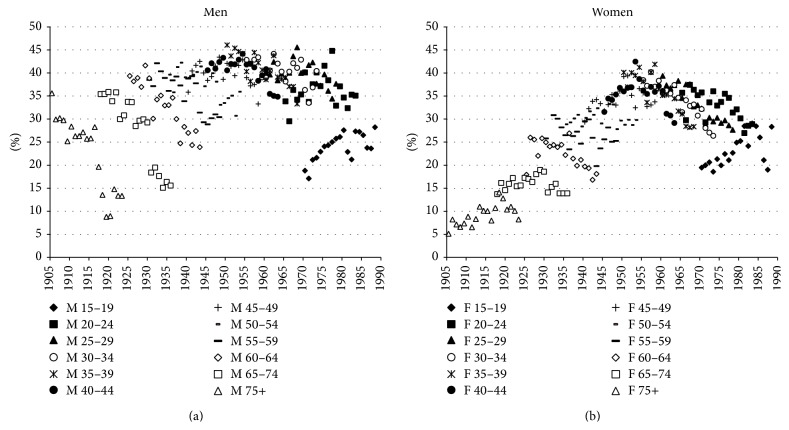
Smoking prevalence by five-year age groups and birth cohort (1905–1990), based on data from calendar year 1988 onwards. Source data: unpublished data Stivoro.

**Figure 8:**
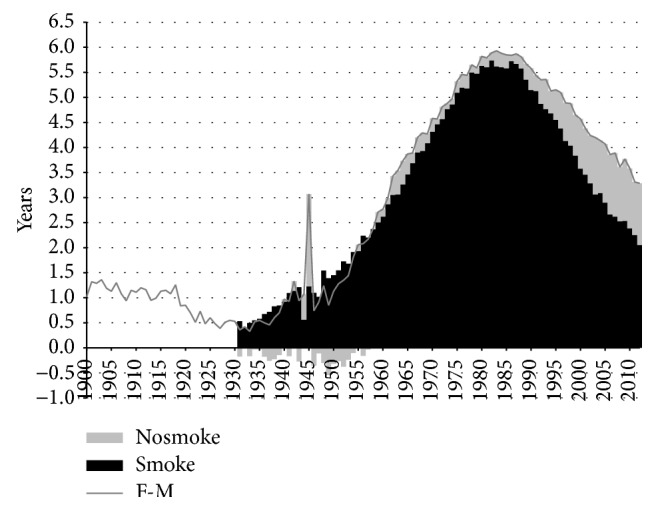
Contribution of smoking- and nonsmoking-related mortality to the difference between men and women in remaining life expectancy at age of 50, in years, Netherlands, 1931–2012. Nosmoke = nonsmoking-related mortality; smoke = smoking-related mortality. M = males; F = females.

**Table 1:** The contribution of different age groups and different causes of death to the sex difference in life expectancy at birth (e0), separately for smoking-related and nonsmoking-related mortality, Netherlands, selected years.

	Absolute contribution (in years)				Relative contribution (in percentage)			
	1931	1950	1983	2012	1931	1950	1983	2012
Sex difference (e0) all-cause mortality	**1.33**	**2.27**	**6.63**	**3.67**	**1.33**	**2.27**	**6.63**	**3.67**
Contribution of smoking-related mortality	0.82	2.05	5.83	2.16	62%	90%	88%	59%
Contribution of nonsmoking-related mortality	0.51	0.22	0.80	1.51	38%	10%	12%	41%

Contribution causes of death								
Mortality from infectious diseases	**0.24**	**0.31**	**0.11**	**0.23**	**18%**	**13%**	**2%**	**6%**
Cancer mortality	**−0.11**	**0.16**	**1.77**	**1.43**	**−8%**	**7%**	**27%**	**39%**
Smoking-related cancer mortality	0.14	0.56	2.46	1.43	11%	25%	37%	39%
Nonsmoking-related cancer mortality	−0.25	−0.40	−0.69	0.00	−19%	−18%	−10%	0%
CVD mortality	**−0.15**	**0.22**	**2.88**	**1.03**	**−11%**	**10%**	**43%**	**28%**
Smoking-related CVD mortality	0.17	0.57	1.67	0.24	13%	25%	25%	6%
Nonsmoking-related CVD mortality	−0.32	−0.35	1.21	0.80	−24%	−16%	18%	22%
Respiratory disease mortality	**0.17**	**0.15**	**0.55**	**0.37**	**13%**	**7%**	**8%**	**10%**
Smoking-related respiratory disease mortality	0.04	0.12	0.57	0.28	3%	5%	9%	7%
Nonsmoking-related respiratory disease mortality	0.13	0.03	−0.02	0.09	9%	1%	0%	3%
External mortality	**0.89**	**0.76**	**0.61**	**0.42**	**67%**	**34%**	**9%**	**12%**
Smoking-related external mortality	0.05	0.12	0.18	0.08	3%	5%	3%	2%
Nonsmoking-related external mortality	0.85	0.65	0.42	0.34	64%	28%	6%	9%
Other	**0.29**	**0.68**	**0.71**	**0.18**	**22%**	**30%**	**11%**	**5%**
Other smoking-related mortality	0.42	0.68	0.95	0.13	31%	30%	14%	4%
Other nonsmoking-related mortality	−0.13	0.00	−0.25	0.05	−10%	0%	−4%	1%

Contribution of age groups								
0	0.90	0.48	0.14	0.05	68%	21%	2%	1%
1–4	0.17	0.09	0.03	0.02	13%	4%	0%	1%
5–19	0.11	0.26	0.15	0.06	9%	12%	2%	2%
20–49	−0.14	0.43	0.74	0.36	−10%	19%	11%	10%
50–64	0.06	0.62	1.89	0.58	4%	27%	28%	16%
65–79	0.19	0.31	3.07	1.63	14%	14%	46%	44%
80+	0.05	0.08	0.61	0.97	4%	3%	9%	27%

**Table 2:** The six main cause-of-death groups used in the study and the related ICD9 codes.

Abbreviation	Description	Numbers within the 65-cause list [22]	ICD9
infect	Infectious diseases (based on the classification by Wolleswinkel-van den Bosch et al. [23])	8–12, 14, 15, 18–22, 24–28, 35–39, 43-44, 50–53, 58, 59	001–004, 006–018, 020–027, 030–057, 060–066, 070–075, 077–088, 090–104, 110–118, 120–139, 320–326, 380–392, 466, 480–487, 510-511, 532, 540–543, 555–558, 562, 567, 580, 670, 681-682

resp	Chronic respiratory diseases	29 + 30	415, 460–465, 470, 472–478, 490–496, 500–508, 512–529

cancer	Cancers	2–6	142, 150–165, 170–175, 179–185, 200, 202, 203

cvd	Cardiovascular disease = cerebrovascular diseases + diseases of circulatory system	13, 32–34	393–398, 401–405, 410–414, 416-417, 420–438, 445, 451–456, 458-459

extern	External causes of death = violence + suicide	61–64	005, 304-305, E800–807, E810–E838, E840–E848, E850–E876, E878–E888, E890–E903, E905–E978, E980–E999

other	Other diseases	rest (1–65)	rest (001–E999)

**(a):** 

	All causes	All causes	Cancers	Cancers	Vascular	Vascular	Respiratory	Respiratory	External	External	Other	Other
	M	F	M	F	M	F	M	F	M	F	M	F
35–39	2.12	1.00	1.00	1.00	4.63	1.15	1.00	1.00	2.22	1.28	2.02	3.85
40–44	2.29	1.03	1.53	1.00	4.07	1.69	1.00	1.12	2.02	1.27	2.01	3.29
45–49	2.40	1.43	2.06	1.26	3.55	2.08	2.23	2.57	1.84	1.26	1.99	2.80
50–54	2.45	1.71	2.46	1.49	3.07	2.33	3.53	3.64	1.69	1.25	1.95	2.39
55–59	2.44	1.89	2.72	1.65	2.63	2.44	4.40	4.33	1.56	1.23	1.91	2.04
60–64	2.36	1.97	2.84	1.74	2.24	2.41	4.84	4.64	1.46	1.22	1.86	1.77
65–69	2.22	1.94	2.83	1.75	1.89	2.24	4.84	4.56	1.38	1.21	1.79	1.57
70–74	2.02	1.79	2.67	1.70	1.59	1.92	4.42	4.11	1.33	1.20	1.72	1.44
75–79	1.76	1.55	2.38	1.57	1.32	1.47	3.56	3.28	1.30	1.18	1.63	1.38
80+	1.44	1.19	1.96	1.37	1.10	1.00	2.27	2.06	1.30	1.17	1.54	1.40

**(b):** 

Cause	Smoothing	Age selection regression	Age selection regression	RR < 1 → RR = 1	RR < 1 → RR = 1
		Men	Women	Men	Women
All causes	Age + age squared	35+	40+; RR40–44 = 1		35–39
Cancers	Age + age squared	35+; RR35–39 = 1	40+; RR40–44 = 1	35–39	35–39, 40–44
Vascular	Age + age squared	35+	40+		80+
Respiratory	Age + age squared	40+	40+	35–39, 40–44	35–39
External	Age + age squared	35+	40+		

## Appendix

1. See Table 2.
2. See Table 3.
3. See Figure 6.
4. See Figure 7.
5. See Figure 8.
